# Evaluating Midazolam’s Influence on Bispectral Index and Propofol Concentrations Using Schnider and Eleveld Models in Target-Controlled Infusion General Anesthesia: A Prospective Observational Study

**DOI:** 10.3390/life15020219

**Published:** 2025-01-31

**Authors:** Federico Linassi, Paolo Zanatta, Matthias Kreuzer, Emma Ciavattini, Christian Rizzetto, Michele Carron

**Affiliations:** 1 Department of Pharmaceutical and Pharmacological Sciences, University of Padua, 35128 Padua, Italy; 2 Department of Anesthesia and Intensive Care, Ca’ Foncello Treviso Regional Hospital, 31100 Treviso, Italy; paolo.zanatta1@aulss2.veneto.it (P.Z.); emma.ciavattini@gmail.com (E.C.); 3 Department of Anesthesiology and Intensive Care, School of Medicine and Health, TUM Universitätsklinikum, Technical University of Munich, 80333 München, Germany; m.kreuzer@tum.de; 4 Department of Breast Oncologic Surgery, Ca’ Foncello Treviso Regional Hospital, 31100 Treviso, Italy; christian.rizzetto@aulss2.veneto.it; 5 Section of Anesthesiology and Intensive Care, Department of Medicine—DIMED, University of Padua, 35122 Padua, Italy; michele.carron@unipd.it

**Keywords:** intravenous anesthesia, infusion pump, bispectral index monitor, propofol, midazolam, anxiolytics, elderly, adverse effects

## Abstract

Background: Midazolam is widely used in clinical anesthesia, but its effects on the Bispectral Index (BIS) and propofol concentration at the effector site (CeP) are underexplored. This study investigates the pharmacodynamic interaction between midazolam and propofol in total intravenous anesthesia (TIVA) with target-controlled infusion (TCI), focusing on Schnider and Eleveld models. Methods: This prospective study included breast surgery patients receiving TIVA-TCI. BIS and CeP were assessed at loss of responsiveness (LoR), during maintenance (MA), and at return of responsiveness (RoR). Incidences of unwanted spontaneous responsiveness (USRE), burst suppression episodes (BSuppE), and postoperative delirium (POD) were recorded. Results: Midazolam premedication significantly reduced propofol doses and CeP at LoR and during MA, without affecting CeP at RoR. In the Schnider model, midazolam reduced total propofol dose, while in the Eleveld model, it lowered BIS at LoR. Unwanted anesthesia events occurred in 36.2% of patients, including USRE (10%), BSuppE (26.2%), and POD (1.2%). BSuppE rates were lower in the Schnider model and reduced in the midazolam group in the Eleveld model. Conclusions: Midazolam premedication influences CeP and BIS in TIVA-TCI, with model-specific variations, optimizing propofol management and improving patient outcomes.

## 1. Introduction

Midazolam is a short-acting hypnotic, often used as premedication for its sedative, anxiolytic, and amnestic properties [1]. It benefits the intraoperative period by inhibiting implicit memory formation and learning [2]. Intravenous midazolam significantly affects Bispectral Index (BIS) values in patients receiving inhalational anesthesia, highlighting its interaction with hypnotic agents at the brain’s gamma-aminobutyric acid (GABA)A receptors [3]. Studies evaluating the influence of midazolam and propofol on the intraoperative EEG spectrum in elderly patients have shown that midazolam, by enhancing GABAergic transmission, increases frontal alpha power during both induction and maintenance of anesthesia with propofol [4]. This effect underscores the synergistic interaction between midazolam and propofol, facilitating anesthesia induction and stabilization [4]. Additionally, midazolam reduces the propofol dose needed to induce sleep [3]. However, these findings are not always consistent. For instance, no significant differences in BIS values were observed in elderly patients receiving midazolam before propofol–remifentanil anesthesia compared to controls, and midazolam did not reduce the propofol dose required to maintain anesthesia [5].

Target-controlled infusion (TCI) is widely used to achieve stable effect-site concentrations of propofol (CeP) during total intravenous anesthesia (TIVA), based on patient-specific physiological parameters [6,7]. In the context of TIVA-TCI, benzodiazepines, such as midazolam, have been shown to influence CeP, leading to lower induction doses of propofol and shorter induction times, although their impact on CeP during maintenance of anesthesia (MA) appears negligible [8]. This inconsistency suggests that midazolam’s effects may vary depending not only on patient characteristics but also on the pharmacokinetic/pharmacodynamic model (hereafter referred to as “model”) used during TIVA-TCI [4,6,7,8].

Models such as Schnider and Eleveld have been developed to accurately dose propofol during TIVA-TCI, accounting for patient characteristics like age and weight [7]. Notable differences in CeP at the loss of responsiveness (LoR), during MA, and return of responsiveness (RoR) have been observed between these two models [9]. Despite their widespread use, limited research has examined how benzodiazepines like midazolam influence these models. Understanding these interactions is clinically significant. A deeper understanding of how midazolam influences propofol pharmacodynamics is crucial not only for optimizing induction and maintenance protocols but also for minimizing potential adverse outcomes, particularly in vulnerable populations such as elderly patients [9,10,11].

This study aimed to address these gaps by evaluating the effects of midazolam premedication on BIS and CeP during TIVA-TCI using Schnider and Eleveld models. BIS was chosen for its reliability as a direct, continuous, and non-invasive measure of hypnotic level, enabling precise quantification of the anesthetic effect of midazolam. Additionally, the study investigated differences in intraoperative adverse sedative events, contributing to a deeper understanding of midazolam’s role in optimizing anesthetic management.

## 2. Materials and Methods

### 2.1. Study Design

The Ethical Committee of Treviso Regional Hospital, Italy, approved this prospective observational study (Approval No. 681/CE Marca), registered at ClinicalTrials.gov (Identifier: NCT05800288); date of registration: 23 March 2023. The study methodologies adhere to the ethical standards of institutional and national research committees and the principles of the 1964 Helsinki Declaration and its amendments. This manuscript complies with the STROBE guidelines for reporting observational studies.

### 2.2. Study Population

Female patients aged 18 and older, scheduled for oncologic breast surgery, were consecutively recruited from 10 April 2023 to 30 September 2023.

**Figure 1:**
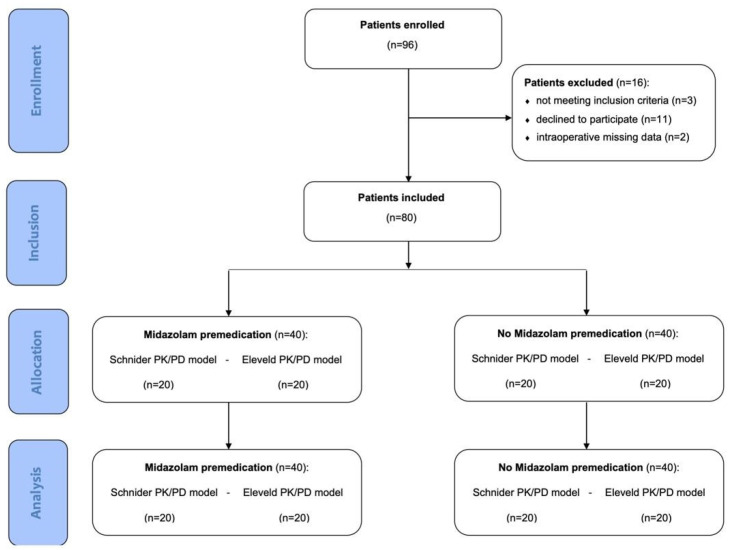
STROBE diagram illustrating the study selection process for the observational study.

Inclusion criteria included patients undergoing TIVA-TCI anesthesia using either the Schnider or Eleveld model and receiving midazolam as premedication. Midazolam was administered by the attending anesthesiologist to patients with state anxiety, identified through the State-Trait Anxiety Inventory (STAI) scale, which was administered pre-operatively by a neuropsychologist [12]. According to the literature, midazolam premedication is defined as the administration of an anxiolytic drug within the operating room prior to anesthesia induction, achieving optimal effects when given 5–20 min before induction [13,14]. Written informed consent was obtained from all participants. The STROBE flow diagram provides further study methodology and participant flow details (Figure 1).

Exclusion criteria included patients with an American Society of Anesthesiologists (ASA) physical status classification exceeding 3 or a body mass index (BMI) of 35 kg/m^2^ or higher. Patients with pre-existing neurodegenerative disorders (e.g., Parkinson’s disease, Alzheimer’s disease), cerebrovascular conditions (e.g., transient ischemic attack, stroke), psychiatric illnesses, chronic respiratory diseases (e.g., COPD, asthma), or cardiovascular issues (e.g., coronary artery disease, arrhythmias, chronic heart failure, peripheral vascular disease) were also excluded. Other exclusion criteria encompassed individuals with renal impairment (e.g., end-stage kidney disease), hepatic dysfunction (e.g., cirrhosis), or those under continuous treatment with antidepressants or anxiolytics. Patients identified with trait anxiety via the State-Trait Anxiety Inventory (STAI) scale, those with a history of alcohol or substance abuse, or those requiring intraoperative vasoactive drugs or neuromuscular blocking agents were not eligible for inclusion

Patient assignment to either the Schnider model group or the Eleveld model group followed a pre-specified alternation based on the 6 h shifts of anesthesiologists participating in the study. This alternation was independent of patient scheduling or characteristics and relied on the anesthesiologist’s familiarity with either the Schnider or Eleveld model during the study period. This approach ensured that allocation was unbiased and mirrored routine clinical practice, as recommended in prior literature [6,9,15].

### 2.3. General Anesthesia

Following an overnight fast, standard vital signs monitoring was initiated before anesthesia induction, including continuous electrocardiogram, pulse oximetry, and non-invasive blood pressure measurement. An intravenous line was placed in the patient’s arm in preparation for TIVA-TCI. A bilateral BIS sensor (Medtronic, Dublin, Ireland) was attached to the patient’s forehead to collect bihemispheric electroencephalogram (EEG) data, which was connected to a BIS XP monitor (Monitor BIS Vista, Version 3.50, Medtronic, Dublin, Ireland) for continuous monitoring. BIS values, which range from 0 (no brain activity) to 100 (awake state), with values between 40 and 60 indicating adequate general anesthesia [16], were continuously recorded.

The surgical pleth index (SPI), derived from photoplethysmographic pulse wave and heartbeat interval analysis, was used to assess hemodynamic responses to surgical stimuli and the nociception–antinociception balance. With an SPI range from 0 to 100, values between 20 and 50 were considered optimal during general anesthesia to obtain adequate analgesia [9].

Anesthesia premedication consisted of an intravenous injection of midazolam at a dosage of 0.03 mg/kg, precisely administered approximately 8–10 min after BIS sensor placement and recording and before the induction of general anesthesia. The preparation process, including the timing of induction, was consistent across all patients, ensuring that the only variable was the administration of midazolam. TIVA-TCI induction and maintenance were performed using CeP and the target concentration at the effect site for remifentanil (CeR). Both were achieved with the uSP6000 syringe pump infusion system (Arcomed AG, Kloten, Switzerland). The Schnider [17] or Eleveld [18] models were employed for propofol administration, while the Minto [19] model was used for remifentanil. Syringes containing 1% propofol and remifentanil (50 ng/mL) were loaded and connected to the patient via a TIVA-TCI giving set.

The initial CeP was set at 1 μg/mL, with increments of 0.5 μg/mL up to 3–4 μg/mL for patients under 50 years of age or 2–3 μg/mL for those over 50, to achieve the LoR, marked by spontaneous eye closure and the inability to follow simple verbal commands. Following the equilibration of the target concentration at the effect site with the plasma concentration, TIVA-TCI was adjusted to maintain a target BIS of 40–60, with CeP adjustments of 0.5 μg/mL at intervals of at least 1 min until the BIS returned to the desired range. CeP during MA (CePMA) was observed after evaluating BIS and SPI five times at one-minute intervals to confirm the steady state.

The starting CeR was set at 0.8 ng/mL before the propofol infusion, with titrations of 0.5 ng/mL every 2 min following the LoR [9]. Adjustments were made to maintain an SPI of 20–50 [9], with modifications of 0.5 ng/mL at intervals of at least 1 min until the target range was reached.

Upon achieving LoR, an iGel^®^ supraglottic airway device (Intersurgical, Wokingham, UK) was gently inserted to secure the airways and enable volume-controlled protective lung ventilation (Primus anesthesia Workstation, Draeger, Telford, PA, USA). At the surgery’s conclusion, ketorolac tromethamine 30 mg, paracetamol 1 g, and ondansetron 4 mg were administered intravenously for pain and postoperative nausea and vomiting (PONV) prophylaxis, respectively.

Subsequently, targeted CeP and CeR levels were reduced to 0. The iGel^®^ supraglottic airway device was removed upon the RoR, evidenced by spontaneous eye-opening and the ability to follow simple commands. The now spontaneously breathing patient was transferred to the post-anesthesia care unit for monitoring. The confusion assessment method (CAM) was applied 15 and 45 min following RoR to screen for potential postoperative delirium (POD).

### 2.4. Variables and Clinical Endpoints

Age (years), BMI (kg/m^2^), ASA physical status classification, CePs (μg/mL) and CeRs (ng/mL) at LoR, during maintenance of anesthesia, and at RoR, duration of propofol infusion (minutes), time from cessation of propofol infusion to RoR, total dose of propofol (mg), and BIS values were considered for the study.

The primary endpoint was to evaluate the impact of midazolam premedication on BIS values at LoR, during MA, and at RoR, initially across the entire patient population and subsequently comparing those who underwent TIVA-TCI using the Schnider and Eleveld models. The secondary endpoints included assessing the impact of midazolam premedication on BIS values at LoR, during MA, and at RoR in adult and elderly patients; evaluating CePs during anesthesia conducted with TIVA-TCI by using both the Schnider and Eleveld models, and verifying the incidence of adverse sedative events with these models. Adverse sedative events included: (i) unwanted spontaneous responsiveness events (USRE), characterized by any involuntary movement (e.g., limb movement) or somatic reaction (e.g., coughing, chewing, grimacing, breathing against the ventilator, or inadequate ventilation due to vocal cord closure) coupled with a significant hemodynamic response (e.g., tachycardia (>100 bpm) and hypertension (mean arterial pressure > 120% of baseline or mean arterial pressure ≥ 100 mmHg)); (ii) burst suppression events (BSuppE), defined as episodes where the burst suppression ratio (BSR) exceeds 5%, indicating at least 3 s of suppressed EEG activity within 1 min; (iii) postoperative delirium (POD) occurrences, identified by CAM 15 or 45 min following RoR.

Data collection was conducted by a data collector who was not involved in administering anesthesia. This individual was tasked with manually recording the aforementioned variables on a paper data-collection form and electronically capturing and storing BIS data, as well as any incidents of unwanted events during anesthesia. Information regarding CePs was obtained directly from the TIVA-TCI system display at the specified time points.

### 2.5. Statistical Analysis

The sample size calculation estimated a mean BIS difference of 7 between the midazolam and no midazolam groups, with a standard deviation of 11 [20], a type I error of 0.05, and a type II error of 0.2 (power = 0.8). This required a sample size of 80 patients, split equally between the midazolam (40) and no-midazolam (40) groups.

Descriptive statistics summarized sample characteristics. Continuous variables were described using median values and interquartile ranges (IQR) or mean values and standard deviations (SD), depending on the normality of the data distribution. Normality was assessed using the Shapiro–Wilk test.

For continuous variables, comparisons between the midazolam and no-midazolam groups were conducted using the two-tailed Mann–Whitney U test for non-normally distributed data or the independent samples *t*-test for normally distributed data. Categorical data were expressed as counts and percentages (%) and analyzed with the Chi-square or Fisher’s exact test, applied when more than 20% of the cells had expected frequencies below 5.

To evaluate differences in CePs across three time points within each group, the Friedman test was applied for non-normally distributed data, while a repeated-measures ANOVA was used for normally distributed data.

To analyze the effects of midazolam and pharmacokinetic models (Schnider or Eleveld) on BIS and CePs, a linear regression analysis was performed, including interaction terms to evaluate potential combined effects of midazolam and model. This analysis assessed both the main effects of midazolam and the pharmacokinetic model, as well as their interactions. A *p*-value of less than 0.05 was considered statistically significant. Statistical analyses were conducted using R version 3.4.0 (21 April 2017).

## 3. Results

A total of 96 women undergoing breast oncologic surgery were enrolled. Sixteen patients were excluded from the study, leaving 80 patients considered for analysis (Figure 1). Demographic characteristics and data about all patients, additionally compared by models (Schnider vs. Eleveld), are detailed in Table S1 in the Supplementary Materials. BIS and CeP values significantly differed across the three time points (LoR, MA, RoR) in the total population of patients and within both Schnider and Eleveld model groups (*p* < 0.001) (Table S1). Unwanted events during anesthesia were observed in 36.2% of all patients, with USRE in 10% and BSuppE in 26.2% of cases. POD was experienced by 1.2% of the enrolled patients, in only one patient not treated with midazolam premedication (Table S1).

Comparing the two model groups, there were no significant differences in demographic characteristics, propofol dose, duration of anesthesia, and time to RoR between the Schnider and Eleveld model groups (Table S1). At LoR, BIS and CeP values were significantly lower in the Eleveld model group than in the Schnider model group (Table S1). During anesthesia maintenance, CeP was significantly higher in the Eleveld model group (Table S1). At RoR, CeP remained significantly higher in the Eleveld group (Table S1). The ΔCeP, defined as the difference between CeP at LoR and CeP at RoR, showed a significant variance between the models, with a notable hysteresis effect only in the Schnider model. No other significant differences were observed (Table S1).

The incidence of total unwanted events during anesthesia was notably higher in the Eleveld model group compared to the Schnider model group, with 60% vs. 12.5% of patients affected, respectively (*p* < 0.001). This difference was particularly evident in the incidence of USRE between the two groups (Table S1).

### 3.1. Effect of Midazolam Premedication in the General Population of Patients

Patients were categorized based on the administration of midazolam premedication, with the control group comprising those who did not receive it. There were no significant differences in demographic characteristics or duration of anesthesia between the two groups (Table 1). The total dose of propofol was significantly higher in the control group, with midazolam premedication leading to a 16.7% reduction in propofol requirements (*p* = 0.013) (Table 1).

**Figure 2:**
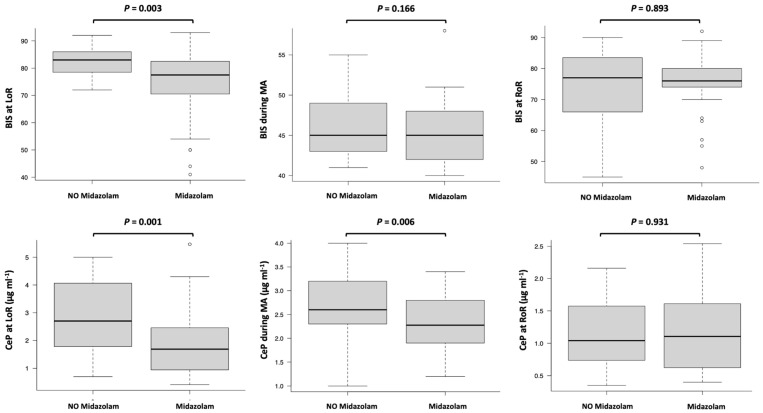
Box plots comparing the effects of midazolam versus no midazolam on the Bispectral Index (BIS) and propofol concentration at the effect site (CeP) during different phases: loss of responsiveness (LoR), maintenance of anesthesia (MA), and return of responsiveness (RoR). Significant differences (*p* < 0.05) are highlighted, indicating the impact of midazolam on BIS and CeP values.

BIS and CeP at LoR, CePMA, CeRMA, and ΔCeP at RoR were all significantly higher in the control group (Table 1, Figure 2). Midazolam premedication resulted in a 40.7% reduction in BIS at LoR (*p* = 0.003), a 6.6% reduction in CeP at LoR (*p* = 0.001), a 15.3% reduction in CePMA (*p* = 0.006), and a 72.2% reduction in ΔCeP at RoR (*p* = 0.004) (Table 1).

No other significant differences were observed (Table 1).

### 3.2. Effect of Midazolam Premedication in the Schnider PK/PD Model

As in the general analysis, there were no significant differences in demographic characteristics or duration of anesthesia between the two groups (Table 1). The total dose of propofol was significantly higher in the control group, with midazolam premedication leading to a 25.4% reduction in propofol requirements (*p* = 0.005) (Table 1).

**Figure 3:**
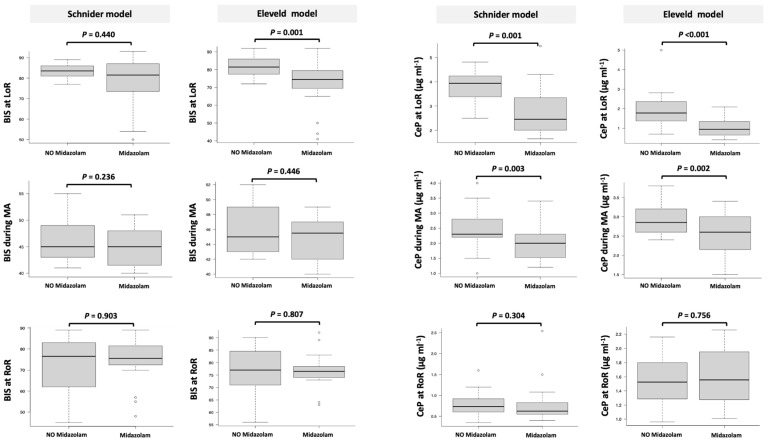
Box plots comparing the effects of midazolam versus no midazolam on the Bispectral Index (BIS) and propofol concentration at the effect site (CeP), analyzed using Schnider and Eleveld models across three phases: loss of responsiveness (LoR), maintenance of anesthesia (MA), and return of responsiveness (RoR). Significant differences (*p* < 0.05) are highlighted, demonstrating variability in BIS and CeP between models and groups.

CeP at LoR, CePMA, CeRMA, and ΔCeP at RoR were all significantly higher in the control group (Table 1, Figure 3).

Midazolam premedication resulted in a 38.4% reduction in CeP at LoR (*p* = 0.001), a 13% reduction in CePMA (*p* = 0.046), and a 37.9% reduction in ΔCeP at RoR (*p* = 0.001) (Table 1).

No other significant differences were observed (Table 1).

### 3.3. Effect of Midazolam Premedication in the Eleveld PK/PD Model

No significant differences were found in demographic characteristics, propofol dose, duration of anesthesia, or time to RoR between the two groups (Table 1).

BIS and CeP at LoR, CePMA, CeRMA, and ΔCeP at RoR were all significantly higher in the control group (Table 1, Figure 3).

Midazolam premedication resulted in an 8.5% reduction in BIS at LoR (*p* = 0.001), a 47% reduction in CeP at LoR (*p* < 0.001), and a 7.1% reduction in CePMA (*p* = 0.040) (Table 1). It also led to a statistically significant reduction in ΔCeP at RoR (*p* = 0.001), although this variable remained relatively stable across groups (Table 1).

BSuppE was significantly lower in the premedication group, with a 69.2% reduction (*p* = 0.001) (Table 1).

Linear regression analysis revealed a significant effect of midazolam on BIS at LoR (*p* < 0.001) and a marginally significant effect of the model used (*p* = 0.051), with no significant interaction between these factors (*p* = 0.16). During anesthesia maintenance, neither midazolam (*p* = 0.143) nor the model (*p* = 0.840) showed significant effects on BIS, and no significant interaction was observed (*p* = 0.977). At RoR, neither midazolam (*p* = 0.635) nor the model (*p* = 0.212) had significant effects on BIS, with no significant interaction (*p* = 0.841). Similarly, significant main effects of midazolam (*p* < 0.001) and the model used (*p* < 0.001) were observed on CeP at LoR, with no significant interaction (*p* = 0.888). For CePMA, significant effects of both midazolam (*p* = 0.003) and the model (*p* < 0.001) were noted, again without significant interaction (*p* = 0.977). At RoR, the model had a significant effect on CeP (*p* < 0.001), while midazolam did not, with no significant interaction (*p* = 0.812).

The boxes represent the median and interquartile range (IQR), with ’whiskers’ extending to the most extreme values within 1.5 times the IQR. Outliers beyond this range are displayed as individual points. Statistical analyses were performed using the Mann–Whitney U test for between-group comparisons, as detailed in the Statistical Methods section.

The figure demonstrates a significant reduction in CeP at LoR and during MA in the midazolam group compared to the no-midazolam group, reflecting midazolam’s sedative and dose-sparing effects on propofol requirements. Additionally, BIS at LoR shows a significant decrease in the midazolam group, supporting its pharmacodynamic impact on hypnotic depth. These findings suggest that midazolam premedication not only reduces propofol dosing but also enhances hypnotic depth, potentially optimizing anesthetic delivery and reducing the risk of adverse effects associated with higher propofol doses.

The figure illustrates significant reductions in CeP at LoR and during MA in the midazolam group compared to the no-midazolam group for both Schnider and Eleveld models. BIS at LoR also demonstrates a significant reduction in the midazolam group, consistent with its sedative effects and pharmacodynamic impact. No significant differences were observed in CeP at RoR. Furthermore, at LoR, CeP was lower in the Eleveld model compared to the Schnider model, while during maintenance and at RoR, CeP was higher in the Eleveld model. The ΔCeP showed a significant variance between models, with a notable hysteresis effect only in the Schnider model. These findings underscore the importance of model selection in optimizing anesthetic depth and minimizing variability, particularly in high-risk or diverse patient populations

## 4. Discussion

Based on this study, midazolam premedication significantly impacts BIS values at LoR and influences anesthesia conduction, as evidenced by variations in CeP values, total propofol dosage, and a reduction in BSuppE with the Eleveld model. The choice between the Schnider and Eleveld models affects BIS, CeP, and the likelihood of adverse events, underscoring the importance of model selection for clinical use [9].

Our findings align with existing literature on midazolam’s impact on BIS at LoR [20], demonstrating that a low dose of midazolam administered within 10 min before anesthesia induction effectively reduces both BIS and CeP at LoR and during MA, but not at RoR.

This underscores midazolam’s role in enhancing hypnosis during the early surgical phase, likely due to its interaction with propofol [21] at GABAA receptors [22,23].

This synergistic interaction may also exert a muscle-relaxant effect, even without neuromuscular blockade, potentially leading to lower BIS values that do not accurately reflect the true sedation or anesthesia level [24]. Variations in BIS effects may result from differences in dosage and timing [21], yet technological advancements have enhanced BIS accuracy by reducing electromyographic artifact interference [25]. However, interactions between low doses of propofol and midazolam may not be fully captured by BIS, potentially leading to misinterpretation of EEG patterns, especially if the specific combination of these drugs is not represented in the BIS database [26]. This could result in the BIS monitor displaying values indicative of a higher level of sedation than actually present until the onset of general anesthesia [27].

Midazolam reduces the required propofol dose for anesthesia induction through synergistic interaction at GABAA receptors, optimizing their clinical effectiveness [22,23]. Administering midazolam before propofol enhances its sedative effect even when given up to 10 min prior [23]. This may account for the lower BSuppE rates observed in premedicated patients using the Eleveld model.

Midazolam premedication significantly enhances patient experience [23] by reducing memory of induction events [22,23], lowering the likelihood of intraoperative implicit memory formation by 65% [9], and avoiding additional complications [22] compared to no premedication. While low oral doses may not significantly affect patient satisfaction [28], appropriate dosing [28] and alternative administration routes, such as intramuscular or intravenous [14,29], provide additional benefits beyond anxiolysis. These include attenuated cardiovascular responses to laryngoscopy and intubation [30,31], reduced PONV [14,30], and improved patient satisfaction [14,29], all without increasing the risk of postoperative delirium [13]. Our findings support the clinical value and safety of low-dose midazolam premedication in elective non-cardiac surgeries with TIVA-TCI [13,32,33].

Midazolam, even when given at low doses for anxiolysis, particularly when combined with other central nervous system depressants like anesthetics and opioids, has been shown to depress respiration by acting on the brainstem and carotid bodies, reducing the ventilatory response to hypoxemia and hypercapnia [34,35,36], thereby raising the risk of respiratory complications post-surgery [37], which should be carefully monitored.

Midazolam is commonly administered intravenously in single doses ranging from 0.5 mg to 4 mg as a sedative, anxiolytic, and amnesic agent before surgery [22,34]. It has a distribution half-life of 6–15 min and an elimination half-life of 1.5–3 h [38]. Its duration of action (60–120 min) with small doses (1–4 mg IV) ensures rapid onset and moderate duration [22,39,40]. These pharmacokinetic properties effectively reduce CePs at LoR and during MA without affecting RoR [38,41]. Small doses of midazolam enhance the effectiveness of low-dose propofol in inducing anesthesia without compromising hemodynamic or respiratory stability [39]. During longer surgeries, as midazolam’s effects wane, propofol dosing may need adjustment to maintain anesthesia depth. Midazolam reduces the required propofol dose through synergistic interaction at GABAA receptors [41], but this effect may also be influenced by changes in drug disposition [42]. Propofol can decrease midazolam clearance by inhibiting hepatic CYP3A4, while midazolam, especially at higher doses, may slow propofol metabolism and clearance due to reduced hepatic perfusion [40,43]. Patient-specific factors, such as hepatic perfusion, enzyme activity (e.g., CYP2B6, CYP2C9), genetic polymorphisms, hypoalbuminemia, age-related changes in clearance, reduced hepatic blood flow due to hypotension, or compromised cardiac output, further influence pharmacokinetic and pharmacodynamic performance. These variables, along with drug interactions, highlight the need for individualized dosing strategies and further PK/PD model validation [15,44].

The differential impacts of the Schnider and Eleveld models on propofol dosing arise from their unique characteristics. The Eleveld model, compared to Schnider, is a general-purpose PK/PD model designed for broader patient populations but with specific limitations [7]. The Schnider model utilizes fixed compartment volumes and adjusts the peripheral fast equilibrating compartment (V2) volume based on age [16]. In contrast, the Eleveld model does not fix values for central and peripheral distribution volumes but instead incorporates demographic variables (age, weight, height, sex) to enhance predictive accuracy [9]. The Eleveld model is noted for better CeP prediction in adults than the Schnider model, although it has shown greater bias in older subjects [45]. Furthermore, a negative correlation between dose and effect, known as the drug titration paradox [46], has been observed with both the Schnider and Eleveld models during stepwise titration toward a target effect [46,47]. However, the Eleveld model, while showing improved CeP prediction accuracy in adults, does not consistently reduce inter-individual variability and may not provide a significant advantage over the Schnider model in certain patient groups [48,49]. Furthermore, the predictive performance of the Eleveld model for BIS values shows significant intra- and inter-subject variability, which limits its reliability for precise BIS-guided titration of propofol during TIVA [48]. Anesthesiologists should consider the pharmacokinetic differences between models to mitigate adverse events, particularly BSupp episodes in older patients using the Eleveld model [12]. Titrating propofol concentrations to achieve adequate anesthesia depth, guided by BIS and clinical monitoring, is crucial during induction. As anesthesia progresses to the maintenance phase, these uncertainties tend to stabilize, aligning the models more closely [12,45]. These differences have significant clinical implications. The Schnider model, with its fixed compartment volumes and reduced variability, may be more suitable for older patients, minimizing the risk of over-sedation and burst suppression [9]. Conversely, the Eleveld model’s adaptability to demographic variables offers broader applicability, particularly in younger or more diverse populations, such as obese or pediatric patients [9,45]. However, its higher intra- and inter-subject variability in BIS prediction may reduce precision during BIS-guided titration in TIVA [45]. Anesthesiologists should base model selection on patient-specific factors, surgical requirements, and the need for precise anesthetic depth monitoring.

This study supports previous findings on premedication omission [10], suggesting that midazolam reduces BSupp episodes in TIVA-TCI with the Eleveld model by enabling lower CePs during induction and early maintenance. Our small sample size, with only one patient (without midazolam premedication) experiencing POD, prevents us from discussing this, but since BSuppE are related with higher POD events [50], and we found that midazolam decreases BSuppE, we can confirm the findings of recent literature regarding the safety of midazolam administration also in frail patients [33]. However, increased USREs observed in the Eleveld group, compared to the Schnider model group, highlight the need for precise CeP titration to balance efficacy and safety. Notably, no significant CeP differences at RoR were found, likely due to midazolam’s transient effect [29], while the hysteresis effect persisted in the Schnider model group [8]. Anesthesiologists should consider these dynamics when managing TIVA-TCI to optimize depth of anesthesia and minimize adverse events, particularly in older patients prone to BSupp with the Eleveld model [9,10].

This study acknowledges several limitations. First, it is observational rather than randomized. Although randomized controlled trials are the gold standard for assessing intervention efficacy, limiting causal inferences, observational studies provide valuable insights, especially when randomization is impractical [51]. Second, our research was limited to female patients undergoing breast surgery, which restricts the generalizability and applicability of the findings to other populations and surgical contexts. While this focused approach allowed for a detailed investigation of pharmacokinetic and pharmacodynamic interactions in a homogeneous population, it does not account for potential variability in other demographics, such as pediatric, obese, or critically ill patients. Future studies should include a broader demographic and assess raw and processed EEG data to fully evaluate anesthesia-related events across diverse clinical scenarios. Third, excluding certain comorbid conditions, common in real-world practice, is a limitation. This was performed to minimize confounders and ensure a more homogeneous population. Fourth, for the sample size was calculated based on the primary endpoint, which may result in limited statistical power to detect significant differences in secondary outcomes, such as adverse events. Additionally, the potential for alpha risk inflation due to multiple comparisons should be considered when interpreting these findings. Fifth, the role of remifentanil deserves attention. Although propofol was the primary agent for maintaining unconsciousness, titrated to optimal BIS values, the impact of remifentanil in attenuating responsiveness—through nociceptive suppression or arousal reduction—should not be underestimated [52]. Sixth, the study did not differentiate between state and trait anxiety, which could influence anesthetic requirements and the effectiveness of midazolam differently [53,54,55]. While midazolam may mitigate the increased propofol requirements associated with preoperative anxiety [53], it is important to note that this effect is more closely related to state anxiety rather than trait anxiety [54,55]. Seventh, a limitation of this study is the scarcity of comparative research evaluating the Schnider and Eleveld models, highlighting the need for further investigations to validate these findings and explore their potential for personalized anesthetic strategies aimed at optimizing patient outcomes and minimizing adverse events [9]. Finally, a comprehensive evaluation of postoperative neurocognitive disorders, considering both pre- and post-operative cognitive status, is essential to affirm midazolam premedication safety, especially in elderly patients [56].

## 5. Conclusions

This study highlights the benefits of midazolam premedication in optimizing propofol anesthesia by reducing propofol requirements during induction and the early maintenance phase. The synergistic effect between midazolam and propofol improves anesthesia management and safety without delaying recovery, even in high-risk patients. The choice of pharmacokinetic model (Schnider or Eleveld) significantly influences outcomes, emphasizing the need to tailor the model to the patient population. Furthermore, our findings underscore the critical role of EEG monitoring, including raw waveforms and spectrograms, in effectively guiding anesthesia. By combining an appropriate model selection with a step-wise patient-specific induction guided by depth-of-anesthesia monitoring, anesthesiologists can minimize adverse events such as unwanted spontaneous responsiveness and burst suppression, thereby enhancing clinical outcomes.

## Figures and Tables

**Table 1:** Comparison of demographic, clinical, and pharmacokinetic variables between midazolam premedication and no-midazolam groups for the total population and stratified by Schnider and Eleveld models.

	Total Population			Schnider Model			Eleveld Model		
Variable	No Midazolam 40 Patients	Midazolam 40 Patients	*p*-Value	No Midazolam 20 Patients	Midazolam 20 Patients	*p*-Value	No Midazolam 20 Patients	Midazolam 20 Patients	*p*-Value
Age, yrs	61 [57, 66]	58 [52.7, 65]	0.125	62.5 [59, 66.2]	58.5 [52.7, 65.2]	0.233	59 [57, 65]	57.5 [52.5, 62.7]	0.336
Age ≥ 65 yrs, n (%)	14 (35)	12 (70)	0.812	8 (40)	7 (35)	1.000	6 (30)	5 (25)	1.000
Weight, kg	68 [62.5, 78.5]	76 [64, 82.2]	0.147	69 [62.7, 78.5]	76.5 [63.5, 83.2]	0.432	67.5 [62.5, 77.5]	75.5 [67.7, 90.5]	0.088
Height, cm	167.5 [162.7, 170.2]	170 [164.7, 173]	0.202	167.5 [160, 170.2]	170 [166.5, 173]	0.190	167.5 [164, 170.2]	168.5 [163.7, 174.2]	0.472
BMI, kg/m^2^	24.1 [22.6, 27.6]	26.3 [23.9, 28.4]	0.244	25.3 [22.6, 27.6]	26.5 [21.9, 28.4]	0.946	24.1 [22.2, 27.5]	26.3 [24.6, 29.1]	0.094
BMI ≥ 30, n (%)	6 (15)	6 (15)	1.000	2 (10)	2 (10)	1.000	4 (20)	4 (20)	1.000
ASA, n (%)									
I	11 (27.5)	6 (15)	0.186	6 (30)	5 (25)	1.000	5 (25)	1 (5)	0.182
II	29 (72.5)	32 (80)		14 (70)	14 (70)		15 (75)	18 (90)	
III	0 (0)	2 (5)		0 (0)	1 (5)		0 (0)	1 (5)	
Propofol total dose, mg	532.9 [417.4, 673.3]	443.7 [347.7, 585.9]	**0.013**	520.5 [461.6, 704]	388.1 [304.1, 510.8]	**0.005**	546.6 [376.5, 628.4]	472.2 [372.9, 598.8]	0.499
Anesthesia time, min	56.5 [47.7, 70]	59 [38.5, 73.7]	351	55.5 [46.2, 70]	41 [32.5, 68.5]	0.064	58.5 [48.7, 65]	60.5 [52.7, 75.5]	0.507
LoR									
BIS baseline	97 [97, 98]	97 [97, 98]	0.873	97 [97, 98]	97 [97, 98]	0.520	97 [97, 98]	97 [96.7, 98]	0.182
CeP at LoR, μg/mL	2.7 [1.8, 4]	1.6 [0.9, 2.4]	**0.001**	3.9 [3.4, 4.2]	2.4 [2, 3.3]	**0.001**	1.7 [1.3, 2.3]	0.9 [0.6, 1.3]	**<0.001**
CeR at LoR, ng/mL	0.8 [0.8, 0.8]	0.8 [0.8, 0.8]	0.317	0.8 [0.8, 0.8]	0.8 [0.8, 0.8]	0.317	0.8 [0.8, 0.8]	0.8 [0.8, 0.8]	0.317
BIS at LoR	83 [78.7, 86]	77.5 [70.7, 82.2]	**0.003**	83.5 [81, 86]	81.5 [75.2, 86.5]	0.440	81.5 [77.7, 86]	74.5 [70.2, 79.2]	**0.001**
**Anesthesia maintenance**									
CePMA, μg/mL	2.6 [2.3, 3.2]	2.2 [1.9, 2.8]	**0.006**	2.3 [2.2, 2.7]	2 [1.5, 2.3]	**0.046**	2.8 [2.6, 3.2]	2.6 [2.1, 3]	**0.040**
CeRMA, ng/mL	3 [3, 3]	2.8 [2.4, 3]	**<0.001**	3 [3, 3]	2.5 [2.4, 3]	**0.003**	3 [3, 3]	2.8 [2.5, 3]	**0.002**
BIS at CePMA	45 [43, 49]	45 [42, 48]	0.166	45 [43, 49]	45 [41.7, 48]	0.236	45 [43, 49]	45.5 [42, 46.5]	0.446
Time to CePMA, min	24.5 [19.7, 33.2]	28 [21.7, 36]	0.248	23.5 [20, 30]	27.5 [21.5, 35]	0.218	26 [18.7, 36]	28.5 [21.7, 36]	0.705
RoR									
CeP at RoR, μg/mL	1 [0.7, 1.5]	1.1 [0.6, 1.6]	0.931	0.7 [0.6, 0.8]	0.6 [0.5, 0.8]	0.304	1.5 [1.2, 1.7]	1.5 [1.2, 1.9]	0.756
CeR at RoR, ng/mL	0.6 [0.5, 0.9]	0.8 [0.5, 0.9]	0.264	0.7 [0.6, 0.9]	0.7 [0.5, 1]	0.655	0.6 [0.5, 0.8]	0.8 [0.7, 0.9]	0.055
BIS at RoR	77 [66, 83.2]	76 [74, 79.5]	0.893	76.5 [62, 83]	75.5 [73.7, 81.2]	0.903	77 [71, 84.2]	76.5 [74, 78.2]	0.807
Time to RoR, min	9.5 [7, 13]	8 [6, 11]	0.058	8.5 [7, 11.2]	8 [6, 11]	0.548	9.5 [7, 12]	8 [5.7, 10.2]	0.308
Δ CeP, μg/mL	1.8 [0.3, 3]	0.5 [−0.7, 1.7]	**0.004**	2.9 [2.6, 3.5]	1.8 [1.4, 2.7]	**0.001**	0.3 [−0.2, 0.9]	−0.7 [−0.9, −0.1]	**0.001**
**Unwanted events**									
USRE, n (%)	3 (7.5)	5 (12.5)	0.712	1 (5)	0 (0)	1.000	2 (10)	5 (25)	0.407
BSuppE, n (%)	14 (35)	7 (17.5)	0.126	1 (5)	3 (15)	0.605	13 (65)	4 (20)	**0.010**

This table highlights key differences in propofol dosing, BIS values, and CeP concentrations across the midazolam and no-midazolam groups during different phases of anesthesia (loss of responsiveness, maintenance, and return of responsiveness). Notable findings include significant reductions in total propofol dose and CeP values in the midazolam group compared to the control group. These reductions suggest a potential for optimizing anesthetic dosing strategies to minimize drug exposure and adverse events. Additionally, burst suppression events were significantly lower in the midazolam group for the Eleveld model, highlighting its role in improving neurological safety. Key statistical tests used include Mann–Whitney U for non-normally distributed continuous variables and Chi-square or Fisher’s exact tests where appropriate for categorical variables. The data are presented as medians [IQR] or counts (%), with *p*-values indicating statistical significance. Abbreviations: BMI—body mass index; ASA—American Society of Anesthesiologists physical status classification; BIS—Bispectral Index; CeP—concentrations at the effect site of propofol; LoR—loss of responsiveness; MA—maintenance of anesthesia; RoR—return of responsiveness; ΔCeP—difference between CeP at LoR and RoR; USRE—unwanted spontaneous responsiveness event; BSuppE—burst suppression event. The Schnider and Eleveld models: pharmacokinetic/pharmacodynamic models for propofol administration during TIVA-TCI.

## Data Availability

Data are available from the corresponding author upon reasonable request.

## Supplementary Materials

The following supporting information can be downloaded at: https://www.mdpi.com/article/10.3390/life15020219/s1, Table S1: SMC1: Descriptive Analysis of Female Patients Undergoing Breast Surgery Across Schnider and Eleveld Models.
