# Supplementary material for: Evaluating Midazolam’s Influence on Bispectral Index and Propofol Concentrations Using Schnider and Eleveld Models in Target-Controlled Infusion General Anesthesia: A Prospective Observational Study
Source: Life (Basel). 2025 Jan 31;15(2):219. doi: 10.3390/life15020219 (PMC11856711; doi:10.3390/life15020219)
Supplement: Supplementary file 1 [file life-15-00219-s001.zip › life-3378663-supplementary.pdf]

Table S1. **SMC1: Descriptive Analysis of Female Patients Undergoing Breast Surgery Across Schnider and Eleveld Models**

**Variables**	**Total patients n. 80**	**Total Schnider model n. 40**	**Total Eleveld model n. 40**	**Models Schnider vs. Eleveld P-value**
Age, yrs	60 [54, 66]	61 [53, 66]	58 [55.5, 65]	0.370
• Age ≥65 yrs, n (%)	26 (32.5)	15 (37.5)	11 (27.5)	0.474
Weight, kg	70 [64, 82]	72.5 [63.5, 82]	69.5 [64.7, 80]	0.885
Height, cm	169 [163.7, 171.2]	170 [163, 171.5]	168 [164, 171.2]	0.888
BMI, kg m^{-2}	25.4 [22.7, 28.3]	26.5 [22.4, 27.9]	25.1 [23.7, 28.4]	0.348
• BMI ≥30, n (%)	12 (15.0)	4 (10)	8 (20)	
ASA, I/II/III, n (%)				
• I	17 (21.2)	11 (27.5)	6 (15)	0.428
• II	61 (76.2)	28 (70)	33 (82.5)	
• III	2 (2.5)	1 (2.5)	1 (2.5)	
Propofol total dose, mg	492.6 [366.9, 598.9]	490.9 [363.7, 586]	513.1 [374, 605.3]	0.683
Anaesthesia time, min	58.5 [43.7, 73]	54 [40, 70]	60 [51, 73.2]	0.121
**LoR**				
BIS baseline	97 [97, 98]	97 [97, 98]	97 [97, 98]	0.153
CeP LOR, µg ml^{-1}	2.2 [1.3, 3.3]	3.3 [2.4, 4.1]	1.3 [0.8, 1.8]	**<0.001**

CeR LOR, ng ml^{-1}	0.8 [0.8-0.8]	0.8 [0.8, 0.8]	0.8 [0.8, 0.8]	0.317
BIS at LoR	81 [76, 85.2]	83 [78.7, 86]	78 [73.7, 82.2]	**0.012**
**Anesthesia maintenance**				
CePMA, µg ml^{-1}	2.5 [2, 3]	2.2 [1.8, 2.5]	2.7 [2.4, 3]	**<0.001**
CeRMA, ng ml^{-1}	3 [2.8-3]	3 [2.5, 3]	3 [2.8, 3]	0.690
BIS at CePMA	45 [43, 48.2]	45 [42, 48.2]	45 [43, 48.2]	0.809
Time to CePMA	26 [20, 34.5]	25 [20, 34]	27.5 [20.5, 36]	0.596
**RoR**				
CeP at RoR, µg ml^{-1}	1.1 [0.7, 1.5]	0.7 [0.5, 0.8]	1.5 [1.2, 1.8]	**<0.001**
CeR at RoR, ng ml^{-1}	0.8 [0.6, 0.9]	0.7 [0.5, 1]	0.8 [0.5, 0.9]	0.758
BIS at RoR	76 [70.7, 83]	75.5 [66, 82.2]	77 [72.7, 83]	0.405
Time to RoR	8 [7, 11]	8 [7, 11]	9.5 [7, 12.2]	0.339
Δ CeP, µg ml^{-1}	1.3 [-0.1, 2.6]	2.6 [1.7, 3.1]	-0.1 [-0.7, 0.3]	**<0.001**
**Unwanted events**				
USRE, n (%)	8 (10)	1 (2.5)	7 (17.5)	**0.057**
BSuppE, n (%)	21 (26.2)	4 (10)	17 (42.5)	**0.002**

The Schnider and Eleveld models: pharmacokinetic/pharmacodynamic models for propofol administration during TIVA-TCI. BMI: body mass index; ASA: American Society of Anesthesiologists physical status classification; BIS: Bispectral Index; CeP: concentrations at the effect site (Ce) of propofol; LoR: loss of responsiveness; MA: maintenance of anaesthesia; RoR: return of responsiveness; Δ CeP: difference between CeP LoR and CeP at RoR; USRE: unwanted spontaneous responsiveness event; BSuppE: burst suppression event.
